# Epigenetic Regulation and Molecular Mechanisms in Cardiovascular Diseases: A Review of Recent Advances and Therapeutic Implications

**DOI:** 10.3390/ijms27020983

**Published:** 2026-01-19

**Authors:** Ewelina Młynarska, Kinga Bojdo, Anna Bulicz, Katarzyna Hossa, Wiktoria Lisińska, Paulina Stasiak, Jacek Rysz, Beata Franczyk

**Affiliations:** 1 Department of Nephrocardiology, Medical University of Lodz, 90-419 Lodz, Poland; 2 Department of Nephrology, Hypertension and Internal Medicine, Medical University of Lodz, 90-549 Lodz, Poland

**Keywords:** epigenetic regulation, cardiovascular disease, molecular mechanisms, therapeutic targets

## Abstract

Cardiovascular diseases (CVDs) remain the leading cause of death worldwide, with growing evidence indicating that epigenetic mechanisms play a central role in their onset and progression. This review provides a comprehensive overview of current knowledge on the epigenetic regulation and molecular mechanisms involved in CVDs, as well as their potential therapeutic implications. The findings demonstrate that DNA methylation, histone modifications, and non-coding RNAs are key regulators of gene expression associated with cardiac hypertrophy, atherosclerosis, myocardial infarction, and heart failure. Interactions between epigenetic alterations and inflammatory or oxidative stress pathways further contribute to endothelial dysfunction and vascular remodeling. Emerging therapeutic strategies targeting these mechanisms, including histone deacetylase inhibitors, DNA methyltransferase inhibitors, and RNA-based therapeutics, show promising cardioprotective effects in experimental and early clinical studies. Overall, this review underscores the significance of epigenetic regulation in cardiovascular pathophysiology and highlights the potential of epigenetic-based interventions as a foundation for precision medicine and novel therapeutic approaches in cardiology.

## 1. Introduction

### 1.1. Epidemiology of Cardiovascular Diseases

Cardiovascular diseases (CVDs) remain the leading cause of mortality worldwide, accounting for an estimated 19.8 million deaths in 2022, or 32% of all global deaths (WHO) [1]. Despite improvements in acute care and prevention, the global burden of CVDs continues to rise, largely due to aging populations, urbanization, and the persistence of modifiable risk factors [1]. According to the Global Burden of Disease Study 2023, CVDs accounted for over 437 million disability-adjusted life years (DALYs), reflecting an increase compared with estimates from a decade earlier [2]. Current projections suggest that by 2050, the number of individuals living with some form of cardiovascular disease will nearly double, reaching approximately 1.14 billion people worldwide [3].

These data underscore that CVDs are not only the dominant global cause of death but also a major contributor to chronic morbidity, health inequality, and economic strain [1].

### 1.2. The Importance of Non-Genetic Factors in Cardiovascular Pathophysiology

While hereditary factors play a role in cardiovascular risk, non-genetic determinants including lifestyle, environmental exposures, and metabolic conditions-constitute the majority of attributable risk [1]. Crucial modifiable risk factors include tobacco use, unhealthy diet, physical inactivity, obesity, hypertension, dyslipidemia, and diabetes mellitus.

Recent large-scale epidemiologic studies emphasize that these environmental and behavioral influences contribute to CVD risk not only through classical biochemical pathways but also via epigenetic mechanisms that regulate gene expression without altering DNA sequence. These mechanisms act as a molecular interface between external exposures and genome function, helping explain why individuals with similar genetic backgrounds can have divergent cardiovascular outcomes [4,5].

### 1.3. Pathophysiological Mechanisms Underlying Cardiovascular Disease

Cardiovascular diseases arise from a complex interplay of structural, cellular, and molecular alterations within the heart and vasculature [6,7]. Key pathophysiological processes include endothelial dysfunction, chronic inflammation, oxidative stress, vascular remodeling, and myocardial fibrosis, which collectively contribute to atherosclerosis, cardiac hypertrophy, and heart failure [6,7]. Dysregulation of lipid metabolism and immune responses further exacerbates vascular injury and plaque formation [6,7]. These mechanisms provide the biological context in which epigenetic modifications operate, influencing disease initiation, progression, and potential response to therapy [6,8]. By understanding these pathways, it becomes possible to identify specific epigenetic targets for therapeutic intervention [6].

### 1.4. Overview of Epigenetic Regulation in Health and Disease

Epigenetics refers to heritable and reversible modifications of chromatin that influence gene activity without changing the DNA sequence. Major mechanisms include DNA methylation, post-translational histone modifications, and the activity of non-coding RNAs (e.g., microRNAs and long non-coding RNAs) [8]. These processes are dynamic and responsive to external cues such as diet, stress, hypoxia, or toxins, thereby linking environmental signals to stable changes in gene expression [6].

In cardiovascular biology, epigenetic remodeling has emerged as a pivotal regulator of vascular homeostasis, endothelial function, cardiac hypertrophy, fibrosis, and inflammation. Several investigations, including candidate-gene methylation studies [8,9] and epigenome-wide association studies [4,5], have identified distinct DNA methylation patterns associated with cardiovascular disease risk. Similarly, cell-specific histone acetylation and methylation changes have been implicated in atherosclerotic lesion formation and vascular smooth muscle remodeling [6,7].

Emerging evidence also highlights the regulatory role of non-coding RNAs in cardiac physiology and pathology. Circulating microRNAs have been identified as potential biomarkers for heart failure and coronary disease [10], while recent studies have uncovered novel long non-coding RNAs (lncRNAs) such as CHKB-DT, which exerts cardioprotective effects in murine and human models [11].

Furthermore, epigenetic dysregulation interacts with clonal hematopoiesis of indeterminate potential (CHIP)—an age-related phenomenon linked to increased cardiovascular mortality through inflammatory and epigenetic pathways [12]. These discoveries collectively indicate that epigenetic mechanisms act as both mediators and biomarkers of cardiovascular disease, bridging the gap between environmental exposure, genetic predisposition, and phenotypic expression.

## 2. Materials and Methods

### Search Strategy and Article Selection

This manuscript was prepared as a narrative review of epigenetic regulation and molecular mechanisms in CVDs.

A literature search was performed in PubMed and supplemented by searches in Scopus and Web of Science to identify relevant experimental and clinical studies. The primary search covered publications from 2015 to 2025, with selected earlier landmark studies included where necessary to provide historical or conceptual context.

Search queries combined MeSH terms (in PubMed, where applicable) and free-text keywords related to epigenetics and cardiovascular disease (e.g., DNA methylation, histone modifications, non-coding RNAs, atherosclerosis, heart failure, myocardial infarction, vascular calcification, and hypertension), using Boolean operators (“AND”, “OR”). Reference lists of key articles were also screened.

Eligible publications included original research articles and selected high-quality reviews published in peer-reviewed journals. Non-English publications, conference abstracts without full text, case reports, editorials, and studies not directly related to epigenetic regulation in cardiovascular disease were excluded. Given the narrative and mechanistic focus of this review, no formal meta-analysis was performed. Where available, contradictory findings were reported alongside consistent evidence.

## 3. Epigenetic Mechanisms in Cardiovascular Biology

Epigenetic mechanisms, encompassing DNA methylation and histone modifications, constitute a crucial, multi-layered regulatory network controlling gene expression and driving the development and progression of CVD.

### 3.1. DNA Methylation

DNA methylation represents a key epigenetic mechanism leading to gene silencing. It involves the covalent attachment of a methyl group to the C5 carbon of the cytosine ring within CpG dinucleotides, resulting in 5-methylcytosine (5mC). CpG islands located in promoter regions of actively transcribed genes usually remain unmethylated; their methylation modifies the local physicochemical properties of the DNA duplex, including the major groove interaction profile, which has consequences for sequence recognition by DNA-binding proteins [13].

A key mechanism of DNA methylation’s effect on gene regulation is the direct inhibition of transcription factor binding. Methylation of CpG dinucleotides within recognition motifs for factors such as CTCF, KLF4, or E2F reduces their binding affinity for the DNA sequence, disrupting the activation and coordination of genes involved in cardiomyocyte differentiation, endothelial function, cellular stress response, and cell cycle control [14]. Concurrently, this action is associated with the competitive binding and recruitment of Methyl-CpG-Binding Domain (MBD) proteins, including MeCP2 and MBD2 [15]. These proteins initiate the assembly of repressive complexes (e.g., NuRD, Sin3A) that recruit histone deacetylases, leading to the deacetylation of core histones and subsequent chromatin condensation. This type of epigenetic repression targets the expression of genes associated with cardiac remodeling, fibrosis, angiogenesis, and inflammatory responses [16].

DNA methylation also impacts nucleosome organization and chromatin accessibility. Increased methylation promotes nucleosome positioning over promoter regions, reducing local chromatin openness, which is manifested by a decrease in active transcription markers such as H3K27ac and H3K4me3 [17]. Consequently, the transcription of genes vital for contractility, energy metabolism, and endothelial homeostasis is suppressed. An additional regulatory layer involves the disruption of spatial genome architecture. Methylation of sequences at TAD boundaries and insulator sites weakens CTCF binding, modifying enhancer–promoter loops and reorganizing regulatory networks critical for ventricular structure development, impulse conduction, and the response to hemodynamic overload [18,19].

Environmental and metabolic signaling pathways integrate with epigenetic control by modulating the activity of DNA Methyltransferases (DNMTs) and TET dioxygenases. Variability in one-carbon metabolism (folate, B12, SAM/SAH ratio), oxidative stress, and inflammatory states condition the hyper- or hypomethylation patterns of genes implicated in atherosclerosis, hypertension, and endothelial dysfunction. In the myocardial context, the dynamics of TET-dependent demethylation and the role of 5-hydroxymethylcytosine (5hmC) are crucial. TET activity, leading to the conversion of 5mC to 5hmC, increases transcriptional plasticity in cardiomyocytes; aberrant 5hmC profiles correlate with heart failure and pathological myocardial remodeling [20].

In the sphere of inflammatory and atherogenic responses, methylation of promoters for transcriptional regulators (e.g., NF-κB, Nrf2) and adhesion genes (ICAM1, VCAM1) modulates endothelial activation, leukocyte adhesion, and the intensity of oxidative stress, accelerating atherosclerotic processes [21]. Developmentally, hypermethylation of promoters for genes such as TBX5, or GATA4 is associated with impaired cardiac morphogenesis, including left ventricular development, and dilated cardiomyopathy phenotypes [22]. Furthermore, changes in methylation at endothelial and vascular smooth muscle cell (SMC)-specific enhancers affect nitric oxide biosynthesis, vascular reactivity, proliferation, and SMC phenotypic switching (contractile ↔ synthetic), which is pertinent to the progression of atherosclerotic lesions. Finally, DNA methylation remains in close coordination with histone modifications, forming epigenetic feedback loops that stabilize long-term gene silencing. The co-occurrence of repressive marks such as H3K9me3 and H3K27me3 alongside histone deacetylation reinforces the repressive chromatin state and sustains transcriptional programs essential for maintaining or disrupting cardiovascular homeostasis [23].

### 3.2. Histone Modifications (PTMs)

The nucleosome, the fundamental structural unit of chromatin, is formed by wrapping DNA around a histone octamer composed of two copies of H2A, H2B, H3, and H4 proteins. The flexible N-terminal domains of histones, commonly referred to as “tails,” protrude from the nucleosome core and represent the main substrate for numerous Post-Translational Modifications (PTMs) [24]. The dynamics of these modifications are strictly controlled by a specialized enzymatic apparatus that functions as the conceptual “writer–eraser–reader” triad, thus establishing the histone code. PTMs are introduced by “writer” enzymes (e.g., Histone Acetyltransferases (HATs), Lysine/Arginine Methyltransferases (KMTs/PRMTs), and kinases), subsequently removed by “erasers” (e.g., deacetylases, demethylases, and phosphatases), and interpreted by “reader” proteins containing domains recognizing specific tags (e.g., bromodomains for acetylated lysines, chromodomains, PHD, MBT, WD40) [25]. The functional outcome of these interactions is the modulation of DNA accessibility and the transcriptional state. PTMs, for instance through charge modification (as in lysine acetylation), influence the physicochemical properties of the histone tails, which in turn modulates the strength of the DNA binding to the nucleosome and nucleosome positioning. Concurrently, histone PTMs create docking platforms for the recruitment of key regulatory complexes, including chromatin remodeling complexes (e.g., SWI/SNF, ISWI, CHD), co-activators/co-repressors, and components of the transcriptional machinery, ultimately determining the local availability of DNA sequences for transcription [26].

#### 3.2.1. Histone Acetylation

Acetylation of lysine residues (primarily on H3 and H4) is catalyzed by HATs (e.g., p300/CBP). This process neutralizes the positive charge of lysines, leading to weakened histone–DNA interactions and increased chromatin accessibility (euchromatin). Acetylated lysines are recognized by bromodomain-containing proteins (e.g., BRD2/3/4), which function as “readers,” stabilizing transcriptional complexes and supporting RNA polymerase II elongation (e.g., via P-TEFb recruitment). H3K27ac serves as a marker for active enhancers and superenhancers, while H3K9ac/H3K14ac denotes active promoters. In the cardiovascular context, changes in endothelial H3K27ac levels on enhancers regulating NOS3 determine nitric oxide (NO) bioavailability and the inflammatory profile, with H3K27ac reduction promoting a pro-inflammatory phenotype. In cardiomyocytes, p300/CBP mediate hypertrophy programs, while histone deacetylases (HDACs) of Class IIa (HDAC4/5/9) inhibit MEF2, limiting hypertrophy. In atherosclerosis, BRD4 enhances NF-κB-dependent gene expression, hence the modulation of acetylation reading by BET inhibitors represents a promising therapeutic strategy [27,28,29,30].

#### 3.2.2. Histone Methylation

Methylation of lysine and arginine residues (mono-, di-, or tri-) is catalyzed by KMTs/PRMTs. This modification does not alter the charge but generates specific docking sites for “reader” proteins (chromodomains, PHD, MBT, WD40), thus determining the recruitment of activating or repressive complexes in a manner dependent on the position and degree of methylation. H3K4me3 on promoters and the combination H3K4me1 + H3K27ac on enhancers are associated with transcriptional activation. In contrast, H3K27me3 (introduced by PRC2/EZH2) and H3K9me2/3 (introduced by SUV39H1/2, SETDB1) mark states of transcriptional repression and heterochromatin. Demethylases (e.g., KDM6A/B for H3K27me3; LSD1/KDM1A for H3K4me1/2) ensure dynamic control. Deregulation of EZH2 in cardiomyocytes is linked to fibrosis. In SMCs, increased H3K27me3 on contractile genes (e.g., ACTA2, MYH11) favors the synthetic phenotype, which is key in atherosclerosis progression. In the endothelium, increased H3K4me3 on adhesion gene promoters (ICAM1/VCAM1) facilitates leukocyte adhesion, and at the developmental level, PRC2/EZH2 controls the transcriptional programs of TBX5/GATA4/HAND1 [31,32,33].

#### 3.2.3. Histone Phosphorylation

Phosphorylation of serine/threonine residues (e.g., H3S10ph, H2AXS139ph/γH2AX) is catalyzed by kinases activated in stress response and cell cycle pathways (e.g., MSK1/2, ATM/ATR, JNK, p38). This process modifies nucleosome dynamics and integrates cellular signaling with the chromatin state, recruiting transcriptional or repair complexes. H3S10ph is associated with immediate early gene activation, while γH2AX is a sensitive marker for DNA Double-Strand Breaks (DSBs), initiating repair cascades. In the cardiovascular context, hemodynamic and oxidative stress activate p38/JNK/MSK kinases, increasing endothelial H3S10ph on inflammatory gene promoters. In the aging heart and heart failure, the accumulation of γH2AX in cardiomyocytes is observed, linked to mitochondrial dysfunction and inflammation. In ischemia/reperfusion models, stress kinases reprogram histone phosphorylation, modulating the expression of cytoprotective genes [34,35,36].

### 3.3. Non-Coding RNA (ncRNA)

Non-coding RNAs (ncRNAs) constitute a critical, multi-layered element of gene expression control at the post-transcriptional, transcriptional, and chromatin levels. Operating in both cis and trans modes, ncRNAs form complex regulatory networks through interactions with proteins, DNA, other RNAs, and the epigenome, modulating processes key to CVD: cell differentiation/phenotypic switching (cardiomyocytes, endothelium, SMCs), inflammation, fibrosis, metabolism, and angiogenesis.

MicroRNAs (miRNAs) (~22 nt) are processed from pri-miRNA (Drosha/DGCR8) to pre-miRNA (Dicer) and incorporated into the RISC complex with AGO2, direct mRNA degradation or translational repression by binding the 3′UTR. In the heart, miR-1/miR-133 regulate contractility homeostasis (their reduction favors hypertrophy), while miR-208/miR-499 control stress programs. miR-21 promotes fibrosis, and miR-29 lowers collagen levels. In the endothelium, miR-126 supports integrity, and miR-92a inhibits angiogenesis. In SMCs, miR-145/miR-143 maintains the contractile phenotype, and their reduction promotes the synthetic phenotype. miR-33 regulates cholesterol transport (ABCA1/ABCG1) [37,38,39,40].

Long non-coding RNAs (lncRNAs) (>200 nt) function as scaffolds, decoys, guides for epigenetic complexes (PRC2, BRG1), or miRNA sponges (ceRNA). In heart failure, Mhrt acts protectively by repressing BRG1. MALAT1 controls endothelial cell migration, and MEG3/ANRIL are linked to atherosclerosis risk (locus 9p21). SENCR supports the SMC contractile phenotype [41,42].

Circular RNAs (circRNAs) are formed by back-splicing, creating nuclease-resistant rings that function primarily as miRNA/protein sponges and transcriptional regulators. CDR1as (ciRS-7) and circ-SLC8A1/circ-Titin modify homeostasis in cardiomyocytes. circANRIL is linked to controlled vascular cell apoptosis [43,44].

ncRNA regulation is integrated into complex networks, such as the miRNA–lncRNA–mRNA axes (ceRNA mechanism), and through crosstalk with the epigenome, where lncRNAs recruit chromatin-modifying complexes (PRC2/EZH2, BRG1) to determine PTM and DNA methylation patterns. ncRNA profiles are strongly modulated by environmental signals (hypoxia, shear stress, hyperglycemia), which is crucial for CVD progression [45,46].

## 4. Epigenetics in Specific Cardiovascular Pathologies

### 4.1. Atherosclerosis and Vascular Inflammation

Atherosclerosis is a chronic inflammatory disorder of the vessel wall, primarily driven by the innate immune response involving myeloid cells such as monocytes and macrophages [47]. Recently, the involvement of epigenetics in atherosclerosis has gained growing recognition. Epigenetic mechanisms include DNA methylation and demethylation, histone methylation and demethylation, histone acetylation and deacetylation, as well as non-coding RNAs. Increasing evidence indicates that these epigenetic processes contribute to both the initiation and progression of atherosclerosis. Notably, epigenetic modifications of DNA and histones are controlled by specific “writers” that add epigenetic marks and “erasers” that remove them, thereby regulating gene expression. Because epigenetic processes are highly dynamic and reversible, they represent promising therapeutic targets for the treatment of atherosclerosis [48]. In patients with chronic kidney disease (CKD), who are prone to cardiovascular complications, a study comparing oxidative stress markers, homocysteine, and global DNA methylation in peripheral blood leukocytes found that individuals with inflammation exhibited global DNA hypermethylation, which was significantly linked to cardiovascular mortality, even after adjusting for age. Similarly, patients with angiographically confirmed coronary artery disease (CAD) showed higher genomic DNA methylation in peripheral lymphocytes compared to controls, with a positive correlation between hypermethylation and plasma homocysteine levels. These findings suggest that atherosclerosis is closely associated with epigenetic alterations, particularly DNA methylation. The initial stage of atherosclerosis involves endothelial cell activation. In an animal model of DBF-induced atherosclerosis, DNMT1 transcription was significantly increased. Details regarding the effects of DNMT1 inhibition are presented later in the article. Further analysis revealed that DBF-induced DNMT1 upregulation occurs via the mTOR/p70S6K signaling pathway, as pretreatment with rapamycin blocked both p70S6K phosphorylation and DNMT1 expression. Additional studies identified flow-sensitive genes, such as cyclin A and connective tissue growth factor, that promoted DNMT1 expression under DBF conditions. Collectively, these findings indicate that endothelial activation and DBF can induce DNMT expression and DNA methylation, altering gene expression and exacerbating atherosclerosis [49]. Studies have shown that DNMT3a can suppress interleukin-13 expression in T helper 2 cells, thereby reducing inflammation. Other research indicates that DNMT3b regulates macrophage polarization and inflammatory responses through promoter DNA methylation. In addition to DNMT-dependent mechanisms, DNA demethylation is mediated by TET enzymes (TET1, TET2, and TET3). Findings suggest that TET1 expression is increased in atherosclerotic plaques, corresponding to DNA hypomethylation. TET2 has been shown to influence vascular smooth muscle cell phenotype, endothelial cell dysfunction, and macrophage-driven inflammation, contributing to the development of atherosclerosis. TET3, in turn, plays a key role in DNA repair and maintaining gene expression stability. Beyond DNA methylation, RNA methylation has emerged as another important epigenetic mechanism in atherosclerosis. Evidence links N6-methyladenosine (m6A) modification in messenger RNA to atherosclerosis risk factors. The m6A methyltransferase METTL3 has been identified as a key regulator responding to hemodynamic stress and mediating inflammatory processes, providing further insight into the epigenetic regulation of atherosclerosis [50].

Previous studies have established the importance of m6A RNA modification in cardiac stress responses, whereas the contribution of other RNA methylations remains less clear. Recent findings indicate that the m5C writer Nsun2 is upregulated in hypertrophied myocardium and plays an important role in cardiac adaptation to stress. Cardiac-specific loss of Nsun2 suppresses stress-induced hypertrophy but accelerates the progression to heart failure. Mechanistically, Nsun2-mediated m5C modification enhances the translation of PRKACA, the catalytic subunit of PKA, thereby sustaining PKA signaling in cardiomyocytes under hypertrophic stress. Similarly to METTL3, Nsun2 is required for maintaining a normal hypertrophic response, although its role becomes critical mainly under severe or prolonged stress, such as advanced aging or pressure overload. Reduced Nsun2 impairs PKA activity, leading to altered cardiomyocyte structure and function, while Nsun2 overexpression aggravates hypertrophy through excessive PKA activation. In conclusion, Nsun2 is a key regulator of cardiac homeostasis during hypertrophic remodeling by promoting PRKACA translation and PKA signaling. Loss of Nsun2 disrupts this pathway and worsens heart failure, highlighting the importance of m5C-dependent post-transcriptional regulation in cardiac stress adaptation [51].

In addition to m6A and m5C, several other RNA modifications have been described, although their roles in cardiovascular biology are generally less well characterized. N1-methyladenosine (m1A) is a reversible RNA modification present in both mRNAs and ncRNAs. In mRNAs, m1A can influence translation efficiency and is recognized by YTH domain–containing proteins, suggesting a regulatory role at the post-transcriptional level. However, current evidence does not directly link m1A methylation to cardiovascular diseases, despite its enrichment in mitochondrial transcripts and its potential importance for mitochondrial function. Another important modification is internal N7-methylguanosine (m7G), which occurs not only at the 5′ cap of mRNAs but also within internal mRNA regions and various ncRNAs. Internal m7G has been shown to enhance translation efficiency and to be dynamically regulated under stress conditions. METTL1-mediated m7G methylation plays a role in vascular development and angiogenesis, and recent studies suggest its therapeutic potential in ischemic vascular diseases. Nevertheless, evidence linking m7G to cardiovascular pathology remains limited [52].

Pseudouridine (ψ) is one of the most abundant RNA modifications and has been associated with heart failure, left ventricular remodeling, and atrial fibrillation, suggesting its potential as a cardiovascular biomarker. Uridylation regulates RNA stability and has been implicated in myocardial ischemia and post-transcriptional gene regulation. Adenosine-to-inosine (A-to-I) RNA editing affects mRNAs, lncRNAs, and microRNAs involved in vascular function, inflammation, and angiogenesis, linking this modification to cardiovascular homeostasis. Other modifications, including RNA cap methylation, N4-acetylcytidine (ac4C), 2′-O-methylation, and tRNA wobble uridine (U34) modifications, mainly influence translation, mitochondrial function, and inflammatory signaling. Together, these findings indicate that multiple RNA modifications contribute to cardiovascular aging and disease, although their mechanisms remain incompletely understood [52].

Building on these findings, growing evidence indicates that epigenetic regulation also plays a crucial role in endothelial function, which is essential in the early stages of atherosclerosis. Endothelial dysfunction is widely recognized as one of the earliest indicators of atherogenesis and is closely associated with oxidative stress and inflammation. Both in vitro and in vivo studies have demonstrated that DNA methylation influences flow-dependent gene expression in endothelial cells. Turbulent blood flow increases promoter methylation of mechanosensitive genes such as KLF3, KLF4, and HoxA5, leading to their reduced expression, while DNMT1-dependent hypermethylation has been observed in endothelial cells exposed to low shear stress. MicroRNAs (miRNAs) further contribute to the regulation of endothelial responses. The anti-inflammatory miR-10a is downregulated under disturbed flow, whereas mechanosensitive miRNAs such as miR-92a, miR-712, and miR-205 are upregulated, promoting endothelial inflammation. In contrast, nuclear miR-126-5p supports endothelial integrity by inhibiting caspase 3 activity. Together, these findings highlight the key role of epigenetic mechanisms in maintaining endothelial homeostasis and their importance in the early development of atherosclerosis [53].

In addition to DNA and RNA methylation, histone modifications also play a significant role in the epigenetic regulation of atherosclerosis. Histone acetylation and deacetylation are controlled by histone acetyltransferases (HATs) and histone deacetylases (HDACs), respectively. Generally, histone acetylation promotes gene transcription. In cardiac tissue, the best-known HATs are p300 and its co-activator, the CREB-binding protein (CBP). Histone acetylation mediated by HATs appears to contribute to a proatherogenic effect. Overexpression of HDAC3, HDAC5, and HDAC7 has been associated with proatherogenic phenotypes, whereas other findings suggest that endothelial HDAC3 may have an atheroprotective role. Overall, individual HDACs have complex, context-dependent effects on atherosclerosis progression, varying by cell type and disease stage. These contrasting results highlight the complexity of HDAC and HAT functions, as their inhibitors can interact with multiple protein families, leading to diverse transcriptional outcomes across different cell types. Therefore, advanced technologies capable of profiling gene expression and histone modifications at the single-cell level are essential to better understand their specific molecular effects during atherogenesis [54]. Atherosclerosis develops preferentially at arterial sites exposed to disturbed blood flow, highlighting the importance of local endothelial regulation. While earlier bulk transcriptomic and epigenomic studies using the partial carotid ligation model identified flow-sensitive genes and DNA methylation changes, they were limited by cellular heterogeneity. The application of single-cell chromatin accessibility profiling (scATAC-seq) has overcome these limitations by enabling cell-type–specific analysis of epigenomic regulation in endothelial cells in vivo [55].

scATAC-seq analyses revealed that endothelial cells are epigenomically heterogeneous even under stable flow conditions and that disturbed flow profoundly remodels chromatin accessibility. Disturbed flow induces the opening of distinct cis-regulatory elements and transcription factor binding sites associated with inflammatory, mesenchymal, and immune-related programs, whereas stable flow maintains accessibility of atheroprotective elements, including KLF2/KLF4-associated motifs. These findings demonstrate that blood flow regulates endothelial gene expression not only transcriptionally but also through dynamic, site-specific epigenomic mechanisms.

Importantly, single-cell chromatin accessibility studies support the concept that endothelial cells from different vascular beds harbor distinct enhancer landscapes, helping to explain why atherosclerosis develops at specific arterial regions rather than uniformly throughout the vasculature. Thus, scATAC-seq provides critical insight into how local hemodynamic forces shape endothelial epigenomic identity and contribute to the site-specific susceptibility to atherosclerosis [55].

Functionally, histone post-translational modifications (PTMs), particularly methylation and acetylation of histones H3 and H4, can be classified as activating or repressive, either promoting or inhibiting transcription of target genes during processes such as macrophage phenotype transition. In general, histone lysine (K) acetylation facilitates gene expression by increasing transcription factor accessibility and is dynamically regulated by histone acetyltransferases (HATs) and deacetylases (HDACs). In contrast, histone lysine methylation can either activate or repress transcription, depending on the specific lysine residue modified and the degree of methylation, under the reciprocal control of lysine methyltransferases (KMTs) and demethylases (KDMs). Although many epigenetic modifiers have been identified to regulate histone PTMs, only a few have been clearly linked to macrophage polarization and function. Traditionally, macrophage polarization has been described as a shift from resting M0 macrophages toward either pro-inflammatory M1 or anti-inflammatory M2 states. However, gene expression profiling suggests an alternative model in which M1 macrophages can be repolarized directly into M2 phenotypes through epigenetic reprogramming at the transcriptional level. Supporting evidence indicates that histone lactylation may facilitate this repolarization by promoting a metabolic shift from glycolysis to oxidative phosphorylation. Similarly, inhibition of nitric oxide (NO) production has been shown to induce M1-to-M2 transition by restoring mitochondrial function, thereby reducing atherosclerotic progression. Nonetheless, M2 macrophages are not entirely protective; for instance, CD163^+^ (M2-like) macrophages can promote intraplaque angiogenesis, increase vascular permeability, and enhance leukocyte infiltration, ultimately contributing to atherosclerosis progression [56]. Within the atherosclerotic plaque microenvironment, macrophages are exposed to a complex array of stimuli—including oxidized lipids, cytokines, signaling molecules, hypoxia, and necrotic cells—that shape their activation state. Transcription factors play a central role in establishing the epigenetic landscape of these macrophages, integrating various atherogenic signals and driving distinct transcriptional programs. This dynamic interplay results in significant functional heterogeneity among macrophage populations, as demonstrated by single-cell RNA sequencing and mass cytometry analyses of atherosclerotic lesions. Moreover, the spatial organization and balance of macrophage subtypes within plaques are key determinants of lesion stability and the clinical outcomes of atherosclerosis [57].

Taken together, these findings emphasize that atherosclerosis is a multifactorial, epigenetically regulated inflammatory disease. Epigenetic mechanisms—encompassing DNA and RNA methylation, histone modifications, and non-coding RNAs—interact to influence endothelial function, vascular smooth muscle behavior, and macrophage polarization. Understanding these interconnected regulatory networks not only provides deeper insight into disease progression but also highlights promising therapeutic opportunities targeting epigenetic pathways to modulate vascular inflammation and plaque stability.

### 4.2. Myocardial Remodeling and Heart Failure

Pathological myocardial remodeling, characterized by cardiomyocyte hypertrophy, apoptosis, and excessive fibrosis, is an adaptive response to chronic stressors such as hypertension, atherosclerosis, myocardial infarction, and metabolic disorders. Over time, it leads to impaired cardiac output and increased risk of heart failure. Therefore, understanding the molecular mechanisms behind this remodeling is crucial for developing new therapeutic strategies.

Recent studies highlight the significant role of epigenetic regulation-particularly DNA methylation and histone modification-in myocardial remodeling. Genome-wide analyses have shown that DNA methylation patterns change markedly in heart failure, including both hypo- and hypermethylation of CpG islands and promoters, depending on the disease context such as dilated or ischemic cardiomyopathy. Histone modifications also play a key role. Histone acetylation by HATs relaxes chromatin and promotes gene transcription, while HDACs condense chromatin and suppress gene expression. Specific HATs and HDACs, such as HDAC2 and HDAC3, have opposing effects on cardiac hypertrophy and cell proliferation. Histone methylation shows residue-dependent effects-H3K4me3 promotes gene activation, whereas H3K9me3 is repressive. During heart failure, changes in H3K4me3 at α- and β-MHC promoters reflect transcriptional reprogramming linked to fetal gene reactivation. Other histone modifications, such as ribosylation mediated by PARP-1, also influence cardiac remodeling. PARP-1 activation promotes hypertrophy and heart failure by interacting with HDACs and chromatin remodelers and by modulating key signaling pathways including ERK1/2 and PI3K-Akt. Conversely, PARP-1 inhibition protects against remodeling. Overall, both DNA methylation and diverse histone modifications cooperatively regulate gene expression patterns driving pathological myocardial remodeling and heart failure progression [58].

Fibrosis is characterized by the excessive accumulation of extracellular matrix (ECM) components and represents a fundamental pathological process contributing to tissue dysfunction across various organs, chronic diseases, and aging. In the heart, myocardial fibrosis is a major factor leading to cardiac dysfunction and failure, serving as a strong predictor of adverse outcomes and mortality. The surplus of structural and matricellular ECM proteins produced by cardiac fibroblasts accumulates between cardiomyocytes (interstitial fibrosis), in areas of cardiomyocyte loss (replacement fibrosis), and around blood vessels (perivascular fibrosis). Despite its significant clinical relevance, the evaluation of myocardial fibrosis through histological analysis is limited due to restricted access to cardiac tissue biopsies [59].

Recent research has increasingly focused on the role of epigenetic mechanisms in myocardial fibrosis (MF), demonstrating that epigenetic regulation significantly influences fibroblast activation, fibrotic gene expression, and extracellular matrix (ECM) deposition through DNA and histone modifications as well as non-coding RNAs.

A key event in MF is the transformation of cardiac fibroblasts (CFs) into myofibroblasts, characterized by elevated expression of α-smooth muscle actin (α-SMA) and collagen I/III. Transforming growth factor-β1 (TGF-β1) suppresses DNMT1 expression and activity, leading to hypomethylation of the α-SMA promoter and increased α-SMA expression after myocardial infarction. In hypoxia-induced fibrosis, overall DNA methylation and DNMT1/DNMT3b levels rise, while inhibition or knockdown of DNMT3b reduces COL1A and α-SMA expression via regulation of HIF-1α. The antifibrotic effects of DNMT inhibitors such as 5-AzadC and RG108, as well as the role of DNMT3a in promoting MF through RASSF1A silencing and Ras/ERK1/2 activation, are explored in greater detail later in this article. The methyl-CpG-binding protein MeCP2, a DNA methylation “reader,” plays a complex role in MF = acting as both a transcriptional repressor and activator. Its overexpression in cardiomyocytes promotes hypertrophy, fibrosis, and contractile dysfunction, whereas cardiac-specific deletion of MeCP2 alleviates these effects. Increased MeCP2 expression, along with α-SMA upregulation, is also observed in other fibrotic organs. Mechanistically, MeCP2 enhances MF progression by repressing DUSP5, leading to ERK1/2 activation, and by inhibiting RASSF1A-mediated suppression of the Ras/ERK1/2 pathway, thereby promoting myofibroblast proliferation and cardiac remodeling [60]. Myofibroblasts play a central role in post-infarction repair by producing ECM, but their excessive activation leads to pathological fibrosis and cardiac stiffening. The F13A1 gene, involved in ECM crosslinking, is regulated by multiple miRNAs, suggesting complex epigenetic control. Histone modifications critically influence fibrotic gene expression. p300-mediated acetylation promotes, while HDAC4 suppresses collagen synthesis. HDAC2 enhances hypertrophy via Akt signaling, whereas sirtuins (SIRT1/3/6) counteract oxidative stress and fibroblast activation through NF-κB, MAPK/ERK, and PI3K/Akt pathways. DNA methylation also contributes to fibrosis. Hypoxia activates HIF-1α, which induces DNMT1/3a/3b and promotes profibrotic gene expression. HIF-1α and related miRNAs (e.g., miR-126) also regulate angiogenesis and endothelial repair. miRNAs further modulate fibrosis and inflammation resolution-miR-26a, miR-29b, and miR-208 suppress collagen genes, while exosomal miRNAs (miR-21-5p, miR-150, miR-182) promote anti-inflammatory macrophage polarization. Other miRNAs, such as miR-19a/b and miR-375, enhance cardiomyocyte proliferation, angiogenesis, and tissue repair through the PDK-1/Akt pathway. During scar maturation, PDGF signaling stabilizes vasculature and regulates collagen deposition, while histone H3K27me3 and CpG methylation silence regenerative pathways like Notch. Overall, epigenetic regulation through DNA methylation, histone modification, and miRNAs orchestrates fibrosis, angiogenesis, and remodeling after myocardial infarction, offering promising therapeutic targets despite current delivery challenges [61].

Another important epigenetic mechanism involved in cardiac remodeling and heart failure is histone modification beyond acetylation and methylation. The BET family of bromodomain proteins binds acetylated chromatin to promote transcription, influencing cardiac hypertrophy, fibrosis, and heart failure. BRD4, in particular, regulates proinflammatory and proatherogenic genes linked to calcification, thrombosis, and lipid metabolism. BET inhibition with JQ1 suppresses cardiomyocyte hypertrophy and cardiac remodeling in experimental models. The potential therapeutic implications of BET inhibition—illustrated by findings from the BETonMACE phase 3 trial—are explored in greater detail later in this article. Histone phosphorylation also contributes to cardiac remodeling. CaMKIIδ mediates H3 serine-10 phosphorylation during pressure overload, promoting hypertrophy, whereas CaMKIIδ deficiency prevents this modification and reduces stress-induced cardiac gene activation. Histone ubiquitination has been implicated in cardiac disease development. Loss of H2B monoubiquitination (H2Bub1) disrupts cardiac development and is linked to congenital heart defects that predispose to heart failure. Conversely, H2A ubiquitination by PRC1 promotes ischemia–reperfusion injury by repressing Hsp27, a cardioprotective protein that reduces ROS and ferroptosis. Inhibiting PRC1 may thus protect against post-ischemic heart failure. Histone sumoylation, though less studied, is associated with gene repression and cardiac outcomes. SUMO1 deletion increases the risk of congenital heart defects, whereas its overexpression prevents hypertrophy and heart failure in experimental models. In contrast, elevated SUMO2/3 expression correlates with cardiomyopathy and heart failure. Overall, phosphorylation, ubiquitination, and sumoylation of histones remain understudied in heart failure but represent emerging epigenetic mechanisms and potential therapeutic targets [62].

Another key epigenetic layer involved in heart failure is the regulation by non-coding RNAs (ncRNAs). Less than 2% of transcribed RNAs encode proteins, while the vast majority are ncRNAs, which play crucial regulatory roles in transcription and translation. Beyond classical ncRNAs such as tRNA and rRNA, ncRNAs are divided into small ncRNAs (<200 nucleotides, including miRNAs and siRNAs) and long ncRNAs (lncRNAs, >200 nucleotides). LncRNAs are particularly important in chromatin remodeling and gene regulation, influencing both transcriptional and post-transcriptional processes. Several lncRNAs have been implicated in cardiac remodeling and heart failure. The mitochondria-derived LIPCAR is elevated in plasma of patients with post-MI left ventricular remodeling, serving as a potential biomarker. Other studies have identified lncRNAs such as H19, which acts as a negative regulator of cardiomyocyte hypertrophy, and Chaer, which promotes hypertrophy by binding to PRC2 and reducing H3K27me3-mediated gene silencing. The lncRNA Mhrt, abundant in the heart, protects against pathological hypertrophy by blocking BRG1 chromatin remodeling activity, while Chast enhances cardiac hypertrophy by inhibiting autophagy through suppression of PLEKHM1. Differential expression of lncRNAs has also been observed between ischemic and non-ischemic failing hearts, and circulating lncRNAs such as NRON and MHRT show promise as heart failure biomarkers. Moreover, lncRNAs can act as miRNA sponges, such as CHRF binding to miR-489, leading to derepression of hypertrophic genes [63].

External factors, including nutrition and the gut microbiome, also modulate cardiac epigenetics. Microbial metabolites like butyrate, acetate, and folate influence histone acetylation and DNA methylation by regulating enzyme activity. Butyrate inhibits HDACs, increasing histone acetylation and exerting anti-inflammatory and potentially cardioprotective effects. Folate and methionine act as methyl donors for SAM production, essential for DNA and histone methylation, while deficiencies alter epigenetic profiles and metabolic health. Choline metabolism by gut bacteria produces TMAO, a metabolite associated with inflammation and heart failure risk, though its direct epigenetic role in the heart remains unclear. Overall, ncRNAs and microbiome-derived metabolites represent emerging epigenetic regulators of cardiac remodeling and heart failure [63].

In summary, pathological myocardial remodeling and fibrosis leading to heart failure are strongly influenced by epigenetic mechanisms. DNA methylation, histone modifications, and non-coding RNAs regulate genes controlling hypertrophy, fibrosis, and inflammation. External factors such as nutrition and gut microbiome metabolites further modulate these processes. Overall, epigenetic regulation represents a key contributor to heart failure and a potential therapeutic target.

## 5. Molecular Mechanisms Linking Epigenetics and CVD

### 5.1. Crosstalk Between Signaling Pathways and Epigenetic Modulators

A growing body of evidence highlights the intricate interplay between cellular metabolism and epigenetic regulation in cardiovascular pathophysiology. Central to this metabolic-epigenetic crosstalk are intermediates of the tricarboxylic acid (TCA) cycle, particularly alpha-ketoglutarate (α-KG), succinate, and fumarate, which directly influence the activity of chromatin-modifying enzymes. α-KG serves as an essential cofactor for the ten-eleven translocation (TET) family of DNA demethylases as well as Jumonji C (JmjC) domain-containing histone demethylases. These enzymes utilize α-KG and Fe(II) to catalyze hydroxylation reactions that remove methyl groups from 5-methylcytosine in DNA or lysine residues on histones, thereby facilitating transcriptional activation of genes involved in cardiomyocyte homeostasis, stress responses, and adaptive remodeling [64]. Conversely, succinate and fumarate act as competitive inhibitors of α-KG–dependent TET and JmjC demethylases. Accumulation of these metabolites, which frequently arises from mitochondrial dysfunction, ischemia, or impaired electron transport, reduces demethylase activity, resulting in hypermethylation of DNA and histones and subsequent transcriptional repression of key cardioprotective genes. This mechanistic axis provides a direct molecular link between impaired mitochondrial energetics and maladaptive epigenetic remodeling in the heart, explaining, in part, how metabolic stress translates into pathological gene silencing. Importantly, this crosstalk is not unidirectional; epigenetic modifications can further influence mitochondrial gene expression and metabolic enzyme activity, creating a feed-forward loop that exacerbates cardiac dysfunction. Understanding this bidirectional communication between metabolic intermediates and epigenetic modulators may uncover novel therapeutic targets, such as TET or JmjC activators, α-KG analogs, or strategies to modulate succinate and fumarate levels, with the potential to prevent maladaptive remodeling, improve electrophysiological stability, and slow the progression of heart failure [65].

**Figure 1:**
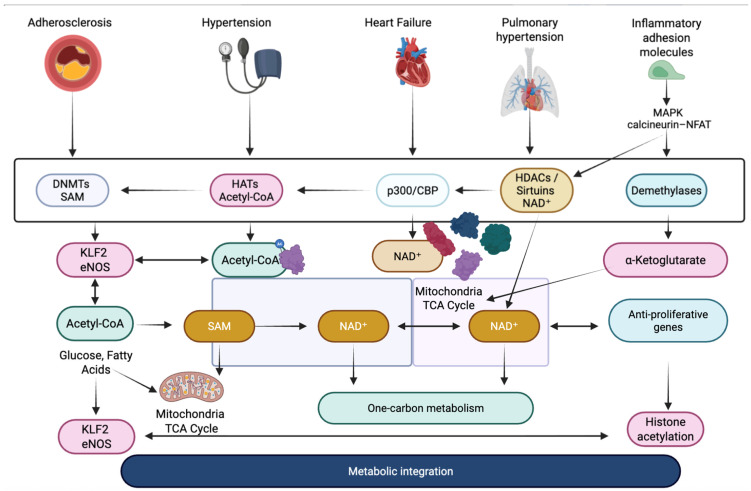
Integration of signaling and epigenetic mechanisms in cardiovascular pathology [61,69,70,71,72,73]. DNMTs—DNA methyltransferases; HATs—histone acetyltransferases; HDACs—histone deacetylases; p300/CBP—histone acetyltransferase coactivators; SAM—S-adenosylmethionine; Acetyl-CoA—acetyl coenzyme A; NAD^+^—nicotinamide adenine dinucleotide; α-ketoglutarate—TCA cycle intermediate; KLF2—Krüppel-like factor 2; eNOS—endothelial nitric oxide synthase; MAPK—mitogen-activated protein kinase; NFAT—nuclear factor of activated T cells; TCA cycle—tricarboxylic acid cycle.

The crosstalk between signaling pathways and epigenetic modulators in cardiovascular disease constitutes a fundamental mechanism by which acute environmental stressors are translated into persistent pathological states across a range of specific conditions, including atherosclerosis, hypertension, heart failure, arrhythmias, and pulmonary arterial hypertension as it shown on Figure 1 [66].

In atherosclerosis, endothelial cells exposed to disturbed shear stress activate MAPK/ERK and NF-κB signaling, which in turn modulates the activity of DNMTs and histone acetyltransferases, leading to hypermethylation of endothelial-protective genes such as *KLF2* and *eNOS* [67]. This epigenetically driven repression amplifies inflammatory adhesion molecule expression, promoting leukocyte recruitment and sustaining plaque development. In vascular smooth muscle cells (VSMCs), angiotensin II–induced activation of the JAK/STAT and PI3K/Akt pathways promotes histone acetylation at proliferation-related genes, facilitating the phenotypic switch from a contractile to a synthetic state, a hallmark of hypertension and restenosis [68]. TGF-β/Smad signaling plays a central role in cardiac fibrosis, especially in heart failure with preserved ejection fraction (HFpEF) and post-myocardial infarction remodeling. Smad complexes directly recruit histone methyltransferases such as EZH2 to promoters of collagen and fibronectin genes, establishing a profibrotic chromatin state that persists even after the initial stimulus has resolved [69]. In cardiomyocytes, pathological hypertrophy driven by chronic β-adrenergic stimulation or pressure overload engages MAPK and calcineurin-NFAT pathways, which cooperate with epigenetic enzymes such as p300/CBP to increase histone acetylation at hypertrophic gene loci, including *ANP*, *BNP*, and *MYH7* [70]. These changes help establish a transcriptional memory of stress that supports long-term hypertrophic remodeling.

In arrhythmogenic conditions, including atrial fibrillation, aberrant oxidative stress and inflammatory signaling converge on chromatin regulators that control ion-channel gene expression. NF-κB activation promotes histone modifications that downregulate potassium and calcium channel genes, destabilizing electrical conduction [70]. In pulmonary arterial hypertension (PAH), dysregulated BMPR2 signaling intersects with DNA methylation changes that silence antiproliferative genes in pulmonary vascular cells, while inflammatory JAK/STAT activity enhances chromatin accessibility at proliferative and antiapoptotic loci [71]. This crosstalk is bidirectional, because epigenetic modifications also modulate signaling pathways. DNA methylation and histone marks determine the expression of angiotensin receptors in hypertension, TGF-β receptors in cardiac fibrosis, and inflammatory cytokine receptors in atherosclerosis. MicroRNAs further shape pathway responsiveness. miR-21 enhances ERK-MAPK signaling in fibrosis, miR-155 amplifies NF-κB activity in atherosclerosis, and miR-208 regulates hypertrophic gene expression in heart failure [72,73].

### 5.2. Epigenetic Feedback Loops

#### 5.2.1. Atherosclerosis

Epigenetic regulation plays a pivotal role in the initiation and progression of atherosclerosis by modulating endothelial function, vascular inflammation, and lipid metabolism. DNA methylation abnormalities in endothelial cells and vascular smooth muscle cells (VSMCs) can trigger pro-inflammatory gene expression, promoting adhesion molecule upregulation and recruitment of immune cells to atherosclerotic plaques. Histone modifications, particularly acetylation and deacetylation mediated by sirtuins (SIRT1) and histone deacetylases (HDACs), regulate nitric oxide synthase (eNOS) activity and the expression of inflammatory mediators like NF-κB, thereby influencing vascular homeostasis [74]. Noncoding RNAs, including lncRNAs such as ANRIL, LEENE, SMILR, and NEXN-AS1, modulate cellular apoptosis, proliferation, and cholesterol efflux, creating feedback loops that either amplify or restrain disease progression. For example, downregulation of NEXN-AS1 in atherosclerotic plaques enhances NF-κB signaling and macrophage recruitment, aggravating lesion development [75]. MiRNAs, including miRNA-143 and miRNA-145, also regulate VSMC phenotype, further reinforcing the epigenetic network that underlies atherosclerosis. Collectively, these interconnected epigenetic mechanisms provide both diagnostic biomarkers and potential therapeutic targets for early intervention and disease management [75,76,77].

#### 5.2.2. Coronary Artery Diseases (CAD)

Coronary artery disease is highly influenced by epigenetic feedback mechanisms that regulate gene expression in endothelial cells, cardiomyocytes, and VSMCs [75]. DNA methylation studies have identified CpG sites associated with genes such as SLC1A5, MPO, and PTCD2, which are involved in amino acid transport, oxidative stress, and lipid metabolism. Abnormal methylation of these regions can disrupt myocardial metabolism, impair glutamine homeostasis, and contribute to coronary plaque development [78,79]. Histone modifications also modulate gene transcription, with acetylation and deacetylation influencing endothelial function and inflammatory pathways. Sirtuins, for example, regulate oxidative stress responses and nitric oxide availability, affecting vascular tone and CAD progression [80]. Noncoding RNAs, including miRNAs like miRNA-143, miRNA-145, and miRNA-106a-3p, influence VSMC phenotypic switching, endothelial inflammation, and plaque stability. LncRNAs such as ANRIL, PANCR, and CAREL further regulate gene expression through chromatin remodeling and miRNA sponging, creating complex feedback loops that govern disease onset and progression. These epigenetic processes not only provide insight into CAD pathophysiology but also identify potential biomarkers for early detection and novel targets for therapeutic intervention [81]. 

#### 5.2.3. Heart Failure

Heart failure (HF) is characterized by maladaptive cardiac remodeling, cardiomyocyte apoptosis, fibrosis, and pathological hypertrophy, all of which are heavily influenced by epigenetic regulation. DNA methylation affects key genes such as DNMT3a, KCNA4, and KCNIP4, altering cardiomyocyte contractile function, mitochondrial metabolism, and electrical conduction [55,81,82]. Histone acetylation, regulated by sirtuins (SIRT1, SIRT3, SIRT6) and HDACs, modulates cardiac hypertrophy, fibrosis, and apoptosis by influencing transcription factors and signaling pathways like TGF-β and SMAD3. For instance, SIRT3 deacetylates GSK3β, which subsequently phosphorylates SMAD3, inhibiting TGF-β-mediated fibrotic responses. Noncoding RNAs, including miRNAs such as miRNA-425, miRNA-744, and miRNA-92b-5p, modulate cardiac fibroblast activation, extracellular matrix deposition, and myocardial remodeling, while lncRNAs like LIPCAR, Meg3, and Wisper regulate fibroblast proliferation and myocardial fibrosis [83]. These epigenetic feedback loops interact to either exacerbate or mitigate heart failure progression, providing both mechanistic insights and potential diagnostic markers or therapeutic targets for improving cardiac function and long-term prognosis [84].

#### 5.2.4. Vascular Calcification

Vascular calcification represents a critical complication of cardiovascular disease, characterized by the phenotypic transformation of VSMCs into osteoblast-like cells. Epigenetic feedback loops are central to this process. DNA methylation changes, such as hypermethylation of miRNA-34b and miRNA-204 and hypomethylation of H19, modulate VSMC differentiation and osteogenic gene expression, including Runx2 and ALP. Histone acetylation and deacetylation through SIRT6, HDAC4, and HDAC9 regulate transcription factors and chromatin accessibility, affecting the expression of osteogenic markers [85]. SIRT6, for example, deacetylates Runx2, promoting its nuclear export and degradation, thereby inhibiting osteogenic differentiation, while HDAC4 and HDAC9 upregulate Runx2, ALP, and osteocalcin, promoting calcification [86]. Noncoding RNAs also contribute: miRNA-30b and miRNA-204 inhibit VSMC osteogenic differentiation, whereas miRNA-128-3p enhances calcification via Wnt signaling activation. These intertwined mechanisms form feedback loops that perpetuate or restrain vascular calcification, offering potential epigenetic targets for prevention and therapy [87].

#### 5.2.5. Myocardial Infarction (MI) and Ischemia–Reperfusion Injury (IRI)

Epigenetic regulation is a key determinant in the onset and recovery from myocardial infarction and ischemia- reperfusion injury. DNA methylation dynamically changes during early stages of MI, affecting genes such as Ptpn6, Csf1r, Col6a1, Cyba, and Map3k14, which are involved in inflammation, oxidative stress, and cellular metabolism. Histone modifications mediated by sirtuins (SIRT1, SIRT3) and HDA. Cs modulate apoptosis, reactive oxygen species production, and mitochondrial permeability, influencing myocardial survival. SIRT1 overexpression, for instance, mitigates reperfusion injury by deacetylating transcription factors involved in oxidative stress response, while HDAC6 exacerbates oxidative damage through deacetylation of peroxiredoxin 1 [88]. Noncoding RNAs, including miRNAs (miRNA-21, miRNA-590-3p, miRNA-199a-3p) and lncRNAs (APF, CAIF, Mirf, UCA1), regulate autophagy, apoptosis, and cardiomyocyte proliferation, forming feedback loops that affect both the severity of injury and the efficiency of cardiac repair. These epigenetic networks are essential for identifying biomarkers and developing targeted therapies for MI and IRI [89].

#### 5.2.6. Hypertension

Hypertension is driven by epigenetic alterations that influence vascular tone, endothelial function, and renin-angiotensin system (RAS) activity. DNA methylation of genes such as mitochondrial fusion 2 and interferon γ promotes VSMC proliferation, oxidative stress, and vascular inflammation, contributing to elevated blood pressure [7,90]. Histone acetylation, regulated by HDACs, sirtuins (SIRT1, SIRT3), and BRD4, affects endothelial nitric oxide production, vasodilation, and vascular remodeling [89]. For example, HDAC6-mediated deacetylation of CSEγ reduces hydrogen sulfide production, exacerbating hypertension. MiRNAs, including miRNA-181a-5p, miRNA-324-5p, and miRNA-34c-5p, modulate RAS activity and endothelial responses, while lncRNAs interact with these pathways to regulate vascular smooth muscle proliferation and inflammation. The interplay between these epigenetic modifications creates feedback loops that amplify or dampen hypertensive responses, providing potential molecular targets for precision therapies [91].

#### 5.2.7. Clonal Hematopoiesis and Epigenetic Inflammation

Clonal hematopoiesis of indeterminate potential (CHIP) is an age-dependent expansion of hematopoietic stem cells carrying somatic mutations in epigenetic regulators such as DNMT3A, TET2, and ASXL1 [12]. Loss-of-function mutations in TET2 impair DNA demethylation, promoting promoter hypermethylation of genes that regulate inflammation and immune responses [12,91]. In murine models, TET2-deficient macrophages exhibit increased activation of the NLRP3 inflammasome and enhanced secretion of pro-inflammatory cytokines such as IL-1β, which drive chronic vascular inflammation and accelerate atherosclerotic lesion formation. Pharmacological inhibition of NLRP3 attenuates these pro-atherogenic effects, supporting a causal role for the inflammasome in CHIP-mediated cardiovascular pathology [92]. Thus, CHIP exemplifies how somatic mutations can induce epigenetic dysregulation of inflammatory signaling, linking hematopoietic genetic alterations to the progression of cardiovascular disease and highlighting potential anti-inflammatory therapeutic targets.

## 6. Therapeutic Implications and Future Directions

### 6.1. Epigenetic Drug Candidates

Recent advances in epigenomics have enabled the development of multiple classes of pharmacological compounds targeting chromatin-modifying enzymes that influence cardiovascular pathology. The most studied groups include inhibitors of DNMTs, HATs, and HDACs, which aim to modulate maladaptive gene-expression programs relevant to cardiovascular disease. Although none of these drugs have yet been approved for use in cardiology, preclinical and translational studies suggest that epigenetic modulation may be a promising strategy for combating inflammation, fibrosis, and adverse cardiac remodeling. Other epigenetic modulators, such as sirtuin activators and bromodomain and extra-terminal (BET) inhibitors, are also being investigated, with most evidence still at the preclinical stage [93].

Among DNMT inhibitors, 5-azacytidine and decitabine are the best characterized. They are nucleoside analogues that form covalent complexes with DNMTs, inducing DNA hypomethylation, and reactivating silenced gene networks. However, their clinical utility is limited by cytotoxicity and poor stability, which has led to the development of newer non-nucleoside inhibitors such as RG108 and GSK-3484862, which show higher selectivity for DNMT1 and fewer side effects. Nevertheless, the lack of tissue selectivity and the risk of whole genome hypomethylation raise serious safety concerns, highlighting the need for more selective compounds, careful dose optimization, and targeted delivery methods [94].

Preclinical studies consistently demonstrate the cardioprotective potential of DNMT inhibition. In pressure overload models, pharmacological inhibition of DNMT activity with RG108 attenuated left ventricular hypertrophy (LVH), reducing the heart-to-body weight ratio from approximately 70% to 47% compared to the control group, reduced myocardial fibrosis, and partially preserved contractile function [95]. In models of ischemic-reperfusion injury, administration of the DNMT inhibitor 5-azacytidine significantly reduced infarct size by approximately 65%, reduced markers of cardiac damage (lactate dehydrogenase (LDH) and creatine kinase (CK)), preserved mitochondrial structure and respiratory function, and restored the activity of antioxidant enzymes such as superoxide dismutase 2 (SOD2), catalase, and glutathione peroxidase 2 (GPx2) [96]. In post-myocardial infarction remodeling, inhibition of DNMT1-dependent hypermethylation prevented miR-133b silencing, thereby reducing fibrotic remodeling and improving myocardial recovery [97].

Human studies further support the relevance of DNMT-associated epigenetic signatures for cardiovascular outcomes, with Mendelian randomization and epigenome-wide association studies (EWAS) linking accelerated epigenetic aging to higher risks of arrhythmias and HF [98]. Chybowska et al. identified DNA methylation-derived protein modules that predicted cardiovascular events independently of traditional risk factors and blood troponin levels, confirming the translational relevance of DNMT-associated epigenetic signatures [99]. EWAS studies conducted in populations with myocardial infarction and coronary artery disease have revealed numerous differences in CpG methylation in genes involved in cardiogenesis, vasculitis, and post-infarction remodeling, such as SOX17, HAND2, WNT7A, MPO, and AHRR, highlighting the role of altered DNA methylation in the progression of cardiovascular diseases [100,101].

Pharmacological inhibition of HDAC has shown consistent cardioprotective effects in preclinical models of acute myocardial infarction (AMI). Broad-spectrum HDAC inhibitors, including trichostatin A (TSA), suberoylanilide hydroxamic acid (SAHA), and valproic acid (VPA), improved ventricular function, reduced infarct size, and attenuated post-ischemic hypertrophy and remodeling in experimental studies [102]. Inhibition of HDAC5 with LMK235 significantly improved cardiac function and prevented abnormal remodeling in a mouse model of pressure overload. Supplementary in vitro data indicated that LMK235 also attenuated angiotensin II (Ang II)-induced cardiomyocyte hypertrophy by regulating the ERK/EGR1-MEF2A pathway, suggesting that HDAC5 may serve as a promising epigenetic target for reducing cardiac hypertrophy [103].

However, the translational applicability of HDAC inhibitors to cardiovascular medicine remains uncertain. Evidence for cardioprotective effects is derived mainly from preclinical work in cultured cardiomyocytes and animal models. Clinical development and oncology use of HDAC inhibitors has highlighted cardiotoxicity as an important consideration, with electrocardiographic abnormalities (especially QT/QTc prolongation) and occasional reports of ventricular arrhythmias, which have informed monitoring and management strategies in oncology practice. In addition, hematological toxicities are recognized as class-associated adverse events. These safety considerations, together with the broad biological actions of HDAC inhibition, currently limit straightforward translation to cardiovascular indications. Further progress will therefore depend on safer and more selective strategies and on carefully designed human studies to better define the benefit–risk profile of HDAC modulation in cardiovascular settings [104].

Additional evidence also points to the cardioprotective potential of HAT inhibition. In experimental animal models, curcumin, a p300 HAT inhibitor, has been shown to prevent hypertension-induced left ventricular hypertrophy and myocardial fibrosis without affecting blood pressure or systolic function. Curcumin treatment reduced cardiac wall thickening, myocyte enlargement, and perivascular fibrosis, accompanied by a reduction in GATA4 transcription factor acetylation. These results highlight the therapeutic importance of p300-dependent acetylation pathways in cardiac hypertrophy and fibrosis, suggesting that modulation of histone acetylation may be a viable anti-remodeling strategy [105].

In addition to inhibiting DNMT, HDAC, and HAT, several other epigenetic strategies are being investigated. These include activation of NAD^+^-dependent deacetylases such as SIRT1, as well as pharmacological targeting of BET proteins. SRT2104, a selective activator of the NAD^+^-dependent deacetylase SIRT1, has shown metabolic and anti-inflammatory benefits in experimental models and may offer new opportunities for cardiovascular protection, although its clinical application remains under investigation [106,107]. Among these emerging approaches, accumulating evidence highlights BET proteins as key epigenetic regulators of stress-induced transcriptional programs in the failing heart.

Under myocardial stress, BRD4, a member of the BET protein family, acts as an acetyl-lysine-recognizing chromatin reader that coordinates the recruitment of transcriptional machinery to regulatory elements controlling inflammatory and profibrotic gene expression. Pharmacological inhibition of BET proteins with small-molecule agents such as JQ1 interferes with bromodomain-dependent chromatin binding, thereby dampening stimulus-driven transcriptional activation and limiting maladaptive remodeling programs [108].

In preclinical models of heart failure, BET inhibition with JQ1 has shown robust cardioprotective effects. In mice exposed to pressure overload induced by transverse aortic constriction, JQ1 attenuated pathological cardiac remodeling, reduced myocardial fibrosis, and preserved ventricular function. Mechanistically, these beneficial effects were attributed to disruption of BRD4-dependent fibroblast–immune cell crosstalk, including suppression of CC-chemokine expression in cardiac fibroblasts and a marked reduction in stress-induced monocyte recruitment to the myocardium [109].

Recent studies extend the therapeutic relevance of BET inhibition beyond pressure overload models to heart failure with HFpEF. In a cardiometabolic HFpEF mouse model, the selective BET inhibitor apabetalone improved diastolic dysfunction, reduced myocardial and systemic inflammation, alleviated lung congestion, and enhanced exercise tolerance. Mechanistically, HFpEF was associated with increased histone acetylation and BRD4 enrichment at pro-inflammatory gene loci, whereas apabetalone suppressed BRD4-dependent inflammatory transcriptional programs, including IL-6–driven signaling [110].

Importantly, the translational relevance of BET inhibition is further supported by clinical data with the BET inhibitor apabetalone, which has been associated with improved lipid profiles, reduced inflammatory markers, and fewer major cardiovascular events in patients with coronary artery disease [107,108,110].

Epigenetic regulation of cardiovascular pathology may also be influenced by widely used cardiometabolic therapies. Sodium-glucose cotransporter-2 (SGLT2) inhibitors have been suggested to indirectly modulate epigenetic signaling pathways in vascular cells, in part through activation of SIRT1. Experimental studies indicate that dapagliflozin may attenuate vascular calcification and pathological vascular remodeling via activation of the SGLT2–SIRT1 axis, an effect associated with SIRT1-mediated deacetylation and transcriptional repression of pro-inflammatory and pro-calcific gene programs, including hypoxia-inducible factor-1α (HIF-1α), a transcriptional regulator implicated in vascular calcification [111]. In parallel, SGLT2 inhibitors have been reported to activate AMP-activated protein kinase (AMPK), which has been associated with suppression of pro-inflammatory transcriptional programs and cytokine signaling in cardiovascular tissues and may indirectly contribute to epigenetic regulation of inflammatory gene expression [112]. Metformin has likewise been reported to influence epigenetic regulation, probably through AMPK-dependent modulation of multiple epigenetic enzymes, including HATs, HDACs, DNMTs, and the class III deacetylase SIRT1. These effects may alter histone acetylation, DNA methylation, and non-coding RNA expression; however, as metformin has been shown to exert both activating and inhibitory effects on distinct epigenetic modifiers, its overall impact on cardiovascular gene regulation appears complex and context dependent [113]. Statins have been shown to modulate epigenetic mechanisms, including DNA methylation, histone acetylation, and non-coding RNA expression, which may partly contribute to their pleiotropic vascular and anti-inflammatory effects beyond lipid lowering [114]. These cardiometabolic therapies, which have been proposed to indirectly influence epigenetic regulation primarily through metabolic and signaling pathways, are summarized in Table 1.

### 6.2. RNA-Based Therapeutic Strategies in Cardiovascular Disease

Small non-coding RNA-based therapeutics have emerged as a promising approach for targeted modulation of gene expression in cardiovascular diseases. The principal therapeutic platforms include antisense oligonucleotides (ASOs), RNA interference-based agents such as short interfering RNAs (siRNAs) and miRNA mimics, as well as RNA aptamers. Among these, anti-miR ASOs, including cholesterol-conjugated antagomiRs, and siRNAs are widely investigated for cardiovascular applications [115].

One clinically evaluated example of miRNA-targeted therapy is CDR132L, a locked nucleic acid (LNA) antisense oligonucleotide directed against miR-132-3p, which is upregulated in human heart failure and contributes to adverse cardiac remodeling. In an early clinical, randomized, placebo-controlled, dose-escalation study in patients with chronic ischemic heart failure, repeated intravenous administrations of CDR132L, were safe and well tolerated, producing a dose-dependent and sustained reduction in circulating miR-132 levels, accompanied by a median decrease in N-terminal pro–B-type natriuretic peptide (NT-proBNP), modest QRS narrowing, and favorable trends in fibrosis-related biomarkers [116].

AntagomiRs are chemically modified antisense oligonucleotides designed to improve nuclease resistance and target affinity through modifications such as phosphorothioate internucleotide linkages, ribose modifications including 2′-O-methyl, 2′-O-methoxyethyl and 2′-fluoro groups, as well as LNA residues. Systemically administered antagomiRs can accumulate in the liver and kidneys and cause hepatotoxicity, thrombocytopenia, or off-target effects, requiring careful preclinical evaluation. To improve delivery to cardiac and vascular tissues, various targeting strategies have been developed, including conjugation of ligands (cholesterol, aptamers, cell-penetrating peptides) and encapsulation in viral vectors, liposomes, extracellular vesicles, or polymeric nanoparticles. Therapeutic translation remains limited by challenges in achieving efficient delivery to cardiovascular cells and by immune responses associated with some delivery platforms [117].

Experimental evidence indicates that selective inhibition of disease-associated miRNAs can modulate several key mechanisms of cardiac pathology. Suppression of the miR-34 family using LNA-modified anti-miRs alleviates adverse ventricular remodeling and improves cardiac function, while targeting miR-1 reduces damage in ischemia–reperfusion models. Inhibition of miR-199a has been shown to alleviate pressure overload-induced hypertrophy, while blockade of miR-379 reduces cardiomyocyte apoptosis in cell and animal studies [118].

Therapies based on siRNAs represent an increasingly important strategy in cardiovascular medicine. These molecules enable catalytic and long-lasting silencing of pathogenic transcripts through RNA interference, and their clinical development has expanded particularly in the context of genetically driven lipid disorders. This mechanism represents post-transcriptional gene silencing in the cytoplasm and does not involve classical epigenetic regulation at the level of chromatin or DNA methylation [119]. A typical example is inclisiran, a siRNA-based drug that suppresses hepatic PCSK9 expression. It has been approved by the US Food and Drug Administration (FDA) and the European Medicines Agency (EMA) as an adjunct to maximally tolerated statin therapy in adults with heterozygous familial hypercholesterolemia (HeFH) or established atherosclerotic cardiovascular disease (ASCVD) who remain above low-density lipoprotein cholesterol (LDL-C) targets. By inhibiting hepatic PCSK9 synthesis, inclisiran increases the availability of LDL receptors in hepatocytes and exerts a sustained LDL-C lowering effect. Owing to its prolonged intracellular activity, the drug requires subcutaneous administration only twice a year [120].

In the ORION-10 study, which included 1561 patients with ASCVD receiving maximally tolerated statin therapy, inclisiran administered on day 1, day 90, and every 6 months thereafter produced a placebo-adjusted LDL-C reduction of 52.3% at day 510 [121]. Similar results were observed in the ORION-11 trial, which included 1617 patients with ASCVD or high cardiovascular risk, confirming the consistency of LDL-C reduction across different populations and lipid-lowering treatment regimens [121]. The ongoing ORION-4 cardiovascular outcomes study (approximately 15,000 participants with pre-existing atherosclerotic cardiovascular disease (ASCVD)) is evaluating whether long-term PCSK9 silencing with inclisiran reduces major adverse cardiovascular events, with completion expected in 2026 [122].

RNA-based therapies continue to face major limitations in cardiovascular medicine, with tissue-specific delivery representing the principal translational bottleneck. To date, the most clinically successful RNA interference-based therapies used in cardiovascular risk reduction rely on liver-directed delivery, exemplified by GalNAc-conjugated agents such as inclisiran, which achieve efficient hepatocyte uptake via the asialoglycoprotein receptor and confer cardiovascular benefit indirectly through hepatic suppression of PCSK9 rather than direct myocardial targeting. In contrast, effective and selective delivery of RNA therapeutics to cardiomyocytes remains an unresolved challenge, as systemically administered delivery platforms lack intrinsic cardiac tropism. In particular, lipid nanoparticles preferentially accumulate in the liver due to reticuloendothelial capture and apolipoprotein-mediated hepatocyte uptake, resulting in limited myocardial exposure. In addition, chemical modifications introduced to improve RNA stability and potency may contribute to off-target effects and immune activation, while delivery platforms (e.g., liposomes, polymers, exosomes, and lipid nanoparticles) continue to face barriers related to cellular uptake and long-term safety, with hepatic or renal toxicity reported in some settings [119,121,123].

### 6.3. Epigenetics-Informed Personalized Medicine

Commonly used cardiovascular risk assessment scales have many well-documented limitations, including age-related misclassification, insufficient external validation, and reliance on traditional risk factors, which reduces their sensitivity and highlights the need for additional biomarkers to improve early detection and risk stratification [124]. DNA methylation-based biomarkers, such as CpG methylation signatures and methylation risk scores (MRS), are becoming a useful complement to existing risk models. By capturing cumulative, long-term exposure to cardiovascular risk factors at the molecular level, DNA-based markers can improve event prediction and enable more precise prognostic stratification when added to clinical algorithms [125]. Clinically, circulating cardiac-enriched miRNAs, particular miR-1, miR-133a/b, miR-208a/b, and miR-499, are elevated in acute coronary syndromes (ACS), while miR-499 also increases in acute heart failure (AHF), reflecting myocardial injury and disease severity and providing candidates for minimally invasive diagnostic and prognostic biomarkers [126].

The expression of non-coding RNA (ncRNA) differs across HF phenotypes, including heart failure with reduced ejection fraction (HFrEF), heart failure with preserved ejection fraction (HFpEF), and heart failure with mildly reduced ejection fraction (HFmrEF) and varies in patients with common comorbidities such as diabetes, CAD, CKD, or AF). These ncRNA patterns, which reflect differences related to HF subtype and comorbidity profile, support molecular stratification, may improve clinical risk stratification, and identify ncRNAs as potential biomarkers and therapeutic targets in HF [127].

Since some cardiovascular and metabolic events occur in people without typical risk factors, new biomarkers are needed to better predict risk. Omic technologies such as genomics, proteomics, and metabolomics enable the assessment of risk dimensions that are largely orthogonal to established clinical factors and therefore may provide incremental improvement when added to existing cardiometabolic disease (CMD) risk scales. However, the high dimensionality and heterogeneity of omics datasets pose a significant obstacle to clinical application, and advanced statistical approaches, including machine learning methods, are being explored to cope with these complex data, while their routine use in practice remains challenging [128].

Beyond traditional cellular biomarkers, epigenetic profiling of cell-free DNA (cfDNA) is emerging as a minimally invasive approach in CVD research. In acute myocardial infarction (AMI), cfDNA exhibits methylation patterns that differ distinctly from genomic DNA (gDNA), with signatures partly derived from cardiac tissue and related to pathways involved in myocardial contraction, inflammation, hypoxia, and lipid metabolism. These methylation signatures reflect ongoing myocardial damage and provide clinically relevant information that can aid in more accurate disease assessment and individualized therapeutic decisions [129].

Recent studies indicate that accelerated epigenetic aging (the discrepancy between biological age and chronological age) is strongly associated with increased mortality in individuals with CVDs. Part of this excess risk appears to reflect underlying vascular and metabolic abnormalities, as elevated angiopoietin-2 levels and diabetes partially mediate these associations. Biological aging markers may therefore help identify patients with accelerated vascular deterioration and improve long-term cardiovascular risk assessment [130]. The key therapeutic classes, mechanisms, representative compounds, and their primary cardiovascular effects are summarized in Table 2.

## 7. Conclusions

Epigenetic regulation plays a fundamental and unifying role in the development and progression of cardiovascular diseases. Across diverse conditions—including atherosclerosis, myocardial remodeling, heart failure, vascular calcification, hypertension, and ischemia–reperfusion injury—DNA methylation, histone modifications, and non-coding RNAs shape gene expression patterns that govern endothelial function, vascular smooth muscle phenotype, inflammatory signaling, fibrosis, and cardiomyocyte adaptation. These mechanisms provide a molecular link between environmental influences, metabolic stress, and lasting pathological changes in cardiovascular tissues.

Key findings demonstrate that specific epigenetic signatures, such as promoter hypermethylation of endothelial-protective genes, histone acetylation-dependent activation of hypertrophic pathways, and ncRNA-driven modulation of inflammatory and fibrotic responses, contribute both to disease initiation and to its long-term persistence through feedback loops that stabilize pathological transcriptional programs. Importantly, these alterations are not static; they show context-dependence across cell types and disease stages, highlighting the dynamic nature of epigenetic remodeling in cardiovascular pathology.

The growing understanding of these mechanisms offers significant translational potential. Epigenetic biomarkers—including circulating microRNAs, lncRNAs, and disease-associated methylation patterns—hold promise for improved risk stratification and early diagnosis. At the therapeutic level, emerging interventions such as BET inhibitors, DNMT and HDAC modulators, and RNA-based therapeutics have demonstrated cardioprotective effects in experimental systems and early clinical investigations. Although challenges remain—particularly in achieving precise tissue targeting and controlling off-target molecular effects—epigenetic-based strategies represent a promising foundation for precision cardiology. As research advances, integrating epigenetic profiling into clinical practice may enable earlier intervention, individualized treatment selection, and novel approaches capable of modifying disease trajectories at the molecular level.

## Figures and Tables

**Table 1:** Indirect epigenetic modulators relevant to cardiovascular disease [111,112,113,114].

Drug Class	Representative Agents	Primary Molecular Targets	Epigenetic-Related Pathways Affected	Epigenetic Effect Type	Cardiovascular Relevance
SGLT2 inhibitors	Dapagliflozin, Empagliflozin	SGLT2	Activation of the AMPK-SIRT1 signaling axis, leading to SIRT1-dependent deacetylation and repression of HIF-1α-driven pro-inflammatory and pro-calcific gene programs.	Indirect epigenetic modulation (histone deacetylation via SIRT1; transcriptional repression)	Attenuation of vascular inflammation, pathological remodeling, and calcification; improvement of endothelial function
Biguanides	Metformin	AMPK	AMPK-dependent regulation of multiple epigenetic enzymes (HATs, HDACs, DNMTs, SIRT1); modulation of histone acetylation, DNA methylation, and non-coding RNA expression	Indirect, context-dependent epigenetic effects	Anti-inflammatory and anti-fibrotic effects; improvement of metabolic-epigenetic coupling in cardiovascular tissues
Statins	Atorvastatin, Rosuvastatin	HMG-CoA reductase; prenylation pathways	Association with changes in DNA methylation patterns; changes in histone acetylation; regulation of miRNA expression	Indirect epigenetic modulation	Pleiotropic vascular and anti-inflammatory effects beyond lipid lowering

**Table 2:** Summary of Epigenetic and RNA-Based Gene Regulatory Strategies in Cardiovascular Disease [93,94,95,96,97,98,99,100,101,102,103,104,105,106,107,108,109,110,111,112,113,114,115,116,117,118,119,120,121,122,123,124,125,126,127,128,129,130].

Therapeutic Category	Gene Regulatory Mechanism (Epigenetic or RNA-Based)	Representative Compounds	Key Reported Effects
DNMT inhibitors	Inhibition of DNA methylation; reactivation of silenced gene networks	5-azacytidine, decitabine, RG108, GSK-3484862	Reduced LV hypertrophy and fibrosis, decreased infarct size, improved mitochondrial function, increased SOD2/catalase/GPx2 activity, restoration of miR-133b after MI
HDAC inhibitors	Inhibition of histone deacetylation; modulation of maladaptive remodeling pathways	TSA, SAHA, VPA, LMK235	Reduced infarct size and hypertrophy, improved LV function; LMK235 modulates the ERK/EGR1-MEF2A pathway in Ang II-induced hypertrophy
HAT inhibitors	Inhibition of histone acetylation mediated by p300	Curcumin (p300 inhibitor)	Prevention of LV hypertrophy and myocardial fibrosis, reduction of GATA4 acetylation
SIRT1 and BET modulators	Regulation of NAD^+^-dependent deacetylation (SIRT1) and bromodomain activity (BET)	SRT2104, apabetalone	SIRT1 activator: metabolic and anti-inflammatory benefits; apabetalone: improved lipid profile, reduced inflammatory markers, fewer cardiovascular events
Anti-miRs/miRNA modulators	Selective inhibition of pathogenic microRNAs	CDR132L, anti-miR-34, anti-miR-1, anti-miR-199a, anti-miR-379	CDR132L: decreased miR-132, reduced NT-proBNP, QRS narrowing, improved fibrosis-related biomarkers; anti-miRs: reduced remodeling, apoptosis, and improved cardiac function
siRNA-based therapy	Post-transcriptional gene silencing via RNA interference (non-epigenetic)	Inclisiran (PCSK9 siRNA)	52% reduction in LDL-C (ORION-10/11), sustained lipid-lowering effect
Epigenetic and RNA biomarkers	CpG methylation signatures, circulating miRNAs, cfDNA methylation, multi-omics profiling	Methylation risk scores (MRS), miR-1/133/208/499, cfDNA-AMI signatures	Improved risk prediction, HF phenotyping, detection of myocardial injury, enhanced AMI assessment

Abbreviations: AMI—acute myocardial infarction, Ang II—angiotensin II, BET—bromodomain and extra-terminal proteins, cfDNA—cell-free DNA, ERK—extracellular signal-regulated kinase, EGR1—early growth response 1, GPx2—glutathione peroxidase 2, HDAC—histone deacetylase, HAT—histone acetyltransferase, HF—heart failure, LDL-C—low-density lipoprotein cholesterol, LV—left ventricle, miR—microRNA, MRS—methylation risk score, NT-proBNP—N-terminal pro-B-type natriuretic peptide, PCSK9—proprotein convertase subtilisin/kexin type 9, SIRT1—sirtuin 1, siRNA—small interfering RNA, SOD2—superoxide dismutase 2, TSA—trichostatin A, VPA—valproic acid.

## Data Availability

No new data were created or analyzed in this study. Data sharing is not applicable to this article.

## Abbreviations

The following abbreviations are used in this manuscript:

5hmC	5-hydroxymethylcytosine
5mC	5-methylcytosine
ABCA1	ATP-Binding Cassette Transporter A1
ABCG1	ATP-Binding Cassette Transporter G1
ACS	Acute Coronary Syndromes
AF	Atrial Fibrillation
AHF	Acute Heart Failure
AHRR	Aryl-Hydrocarbon receptor repressor
AKT	Protein Kinase B
α-SMA	Alpha-Smooth Muscle Actin
AMI	Acute Myocardial Infarction
Ang II	Angiotensin II
ANP	Atrial Natriuretic Peptide
anti-miR	Anti-microRNA Inhibitor
ASCVD	Atherosclerotic Cardiovascular Disease
ASO	Antisense Oligonucleotide
ATM	Ataxia Telangiectasia Mutated
ATR	ATM- and Rad3-Related
BET	Bromodomain and Extra-Terminal Proteins
BMPR2	Bone Morphogenetic Protein Receptor Type 2
BNP	B-type Natriuretic Peptide
BRD2/3/4	Bromodomain-Containing Proteins 2/3/4
BRG1	Brahma-Related Gene-1
CAD	Coronary Artery Disease
CaMKIIδ	Ca2+/Calmodulin-Dependent Protein Kinase II Delta
CBP	CREB-Binding Protein
ceRNA	Competing Endogenous RNA
CFs	Cardiac Fibroblasts
cfDNA	cell-free DNA
CHD	Chromodomain Helicase DNA-Binding
CHIP	Clonal Hematopoiesis of Indeterminate Potential
CK	Creatine Kinase
CKD	Chronic Kidney Disease
CMD	Cardiometabolic Disease
CVD	Cardiovascular Disease
COL1A	Collagen Type I Alpha Chain
CTCF	CCCTC-Binding Factor
DALY	Disability-Adjusted Life Year
DBF	Disturbed Blood Flow
DNMT	DNA Methyltransferase
DNMT1/3a/3b	DNA Methyltransferase 1/3A/3B
DSB	Double-Strand Break
DUSP5	Dual-Specificity Phosphatase 5
ECM	Extracellular Matrix
EGR1	Early growth response 1
EMA	European Medicines Agency
eNOS	Endothelial Nitric Oxide Synthase
ERK	Extracellular Signal-Regulated Kinases
ERK1/2	Extracellular Signal-Regulated Kinases 1/2
EWAS	Epigenome-Wide Association Study
EZH2	Enhancer of Zeste Homolog 2
FDA	Food and Drug Administration
F13A1	Coagulation Factor XIII A Chain
gDNA	Genomic DNA
GPx2	Glutathione Peroxidase 2
GSK3β	Glycogen Synthase Kinase 3 Beta
γH2AX	Phosphorylated H2A Histone Family Member X
H2AX	H2A histone family member X
H2Bub1	Monoubiquitinated H2B
H3K27ac	Acetylation of histone H3 lysine 27
H3K27me3	Trimethylation of histone H3 lysine 27
H3K4me1/2	Mono-/dimethylation of histone H3 lysine 4
H3K4me3	Trimethylation of histone H3 lysine 4
H3S10ph	Phosphorylated Histone H3 at Serine 10
HAT	Histone Acetyltransferase
HDAC	Histone Deacetylase
HeFH	Heterozygous Familial Hypercholesterolemia
HF	Heart Failure
HFmrEF	Heart Failure with Mildly Reduced Ejection Fraction
HFpEF	Heart Failure with Preserved Ejection Fraction
HFrEF	Heart Failure with Reduced Ejection Fraction
HIF-1α	Hypoxia-Inducible Factor 1-Alpha
ICAM1/VCAM1	Intercellular/Vascular Cell Adhesion Molecule 1
IRI	Ischemia–Reperfusion Injury
ISWI	imitation switch chromatin-remodeling complex
JAK/STAT	Janus Kinase/Signal Transducer and Activator of Transcription
JNK	c-Jun N-terminal Kinase
KDM	Histone Demethylase
KLF2/3/4	Krüppel-Like Factor 2/3/4
KMTs	Histone Lysine Methyltransferases
LDH	Lactate Dehydrogenase
LDL-C	Low-Density Lipoprotein Cholesterol
LNA	Locked Nucleic Acid
lncRNA	Long Non-coding RNA
LV	Left Ventricle
LVH	Left Ventricular Hypertrophy
MACE	Major Adverse Cardiovascular Events
MAPK	Mitogen-Activated Protein Kinase
MBD	Methyl-CpG-Binding Domain
MeCP2	Methyl-CpG-Binding Protein 2
METTL3	Methyltransferase-Like 3
MF	Myocardial Fibrosis
MHC	Myosin Heavy Chain
MI	Myocardial Infarction
miR	MicroRNA
MPO	Myeloperoxidase
MRS	Methylation Risk Score
m6A	N6-methyladenosine
MSK1/2	Mitogen- and Stress-Activated Kinase 1/2
NAD^+^	Nicotinamide Adenine Dinucleotide
ncRNA	Non-coding RNA
NF-κB	Nuclear Factor Kappa B
NO	Nitric Oxide
NT-proBNP	N-terminal pro–B-type Natriuretic Peptide
PAH	Pulmonary Arterial Hypertension
PARP-1	Poly(ADP-Ribose) Polymerase 1
PCSK9	Proprotein Convertase Subtilisin/Kexin Type 9
PDGF	Platelet-Derived Growth Factor
PI3K/AKT	Phosphoinositide 3-Kinase/AKT kinase pathway
PLEKHM1	Pleckstrin Homology Domain-Containing Protein 1
PRC1	Polycomb Repressive Complex 1
PRC2	Polycomb Repressive Complex 2
PRMT	Protein Arginine Methyltransferase
PTMs	Post-Translational Modifications
RAS	Renin–Angiotensin System
RASSF1A	Ras Association Domain Family Member 1 Isoform A
RISC	RNA-Induced Silencing Complex
ROS	Reactive Oxygen Species
SAHA	Suberoylanilide Hydroxamic Acid
SAH	S-Adenosyl-Homocysteine
SAM	S-Adenosyl-Methionine
siRNA	Small Interfering RNA
SIRT1/3/6	Sirtuin 1/3/6
SMAD3	Mothers Against Decapentaplegic Homolog 3
SMC	Smooth Muscle Cell
SOD2	Superoxide Dismutase 2
STAT	Signal Transducer and Activator of Transcription
SUMO	Small Ubiquitin-Like Modifier
TAD	Topologically Associating Domain
TBX5	T-box transcription factor 5
TET	Ten–Eleven Translocation Dioxygenase
TET1	Ten-Eleven Translocation Dioxygenase 1
TET2	Ten-Eleven Translocation Dioxygenase 2
TET3	Ten-Eleven Translocation Dioxygenase 3
TGF-β	Transforming Growth Factor Beta
TGF-β1	Transforming Growth Factor Beta-1
TMAO	Trimethylamine N-oxide
TSA	Trichostatin A
UTR	Untranslated Region
VCAM1	Vascular Cell Adhesion Molecule 1
VPA	Valproic Acid
VSMC	Vascular Smooth Muscle Cell
WHO	World Health Organization
